# Inflammatory microRNA-194 and -515 attenuate the biosynthesis of chondroitin sulfate during human intervertebral disc degeneration

**DOI:** 10.18632/oncotarget.17571

**Published:** 2017-05-02

**Authors:** Bo Hu, Chen Xu, Ye Tian, Changgui Shi, Ying Zhang, Lianfu Deng, Hongyu Zhou, Peng Cao, Huajiang Chen, Wen Yuan

**Affiliations:** ^1^ Department of Spine Surgery, Changzheng Hospital, Second Military Medical University, Shanghai, China; ^2^ Shanghai Key Laboratory for Bone and Joint Diseases, Shanghai Institute of Orthopaedics and Traumatology, Shanghai Ruijin Hospital, Shanghai Jiao Tong University School of Medicine, Shanghai, China

**Keywords:** intervertebral disc degeneration, nucleus pulposus, chondroitin sulfate, biosynthesis, glycosyltransferases

## Abstract

Intervertebral disc degeneration (IDD) is characterized by dehydration and loss of extracellular matrixes in the nucleus pulposus region. Chondroitin sulfate has been found to be the water-binding molecule that played a key role in IDD. Although investigators have reported that inflammatory cytokines are involved in the reduction of chondroitin sulfate in IDD, but the underlying mechanism is unrevealed. Since chondroitin sulfate synthesis is controlled by chondroitin sulfate glycosyltransferases CHSY-1/2/3 and CSGALNACT-1/2, their functional role and regulatory mechanism in IDD is not fully studied. Here, we set out to investigate the function and regulatory roles of these factors during IDD development. We found that among these chondroitin sulfate glycosyltransferases, CHSY-1/2/3 are significantly down-regulated in severe IDD samples than mild IDD samples. *In vitro* experiments revealed that Interleukin-1β and Tumor Necrosis Factor-α stimulation led to significant reduction of CHSY-1/2/3 at protein level than mRNA level in NP cells, indicating a post-transcriptional regulatory mechanisms are involved. By computational prediction and analysis, we found that inflammatory cytokines stimulated microRNA-194 and -515 target CHSY-1/2/3 mRNA and significantly interrupt their translation and downstream chondroitin sulfate deposition. Inhibition of microRNA-194 and -515 however, significantly rescued CHSY-1/2/3 expressions and chondroitin sulfate deposition. These findings together demonstrated a vital role of inflammatory stimulated microRNAs in promoting intervertebral disc degeneration by interrupt chondroitin sulfate synthesis, which may provide new insights into the mechanism and therapeutic approaches in IDD.

## INTRODUCTION

Chronic low back pain (CLBP) is a general muscular disorder causing severe social and economic burdens and loss of work. Current studies revealed that CLBP is mostly associated with intervertebral disc degeneration (IDD) [1, 2]. IDD is characterized by a series of pathogenic processes including cellular, biochemical and structural impairment which result in metabolic imbalances of the extracellular matrix (ECM), which mainly take part in the nucleus pulposus (NP) [3–5]. This metabolic disorders lead to the loss of water content and the boundary between annulus fibrosus (AF) and NP. Sequentially these changes caused reduction in disc height and the capability of withstanding mechanical load [6, 7], and ultimately trigger CLBP.

In normal NP, the major structural components of the ECM are type II collagen and proteoglycans. Aggrecan is the most essential proteoglycan component which contains a large number of chondroitin sulfate (CS) chains [8], and conferring gel-like properties on the disc which are responsible for water retention and sustaining the mechanical load [9]. CS is a glycosaminoglycan (GAG) which is composed of repeating disaccharide residues, N-acetylgalactosamine (GalNAc) and glucuronic acid (GlcUA) with sulfate residues binding to the membrane of the Golgi apparatus [10]. These residues are polyanionic due to the high content of sulfate groups, thus they attract and bind water molecules, hydrating the disc [11]. Investigators have identified several glycosyltransferases that are involved in CS biosynthesis: chondroitin sulfate synthase 1 (CHSY-1) [12], chondroitin sulfate synthase 2 (CHSY-2) [13], chondroitin sulfate synthase 3 (CHSY-3) [14], chondroitin sulfate N-acetylgalactosaminyltransferase 1 (CSGALNACT1) [15], and chondroitin sulfate N-acetylgalactosaminyltransferase 2 (CSGALNACT2) [16]. These CS glycosyltransferases function to initiate the synthesis and elongation of CS chain.

Early studies showed that CS content is reduced significantly in severe IDD [17, 18]. Since aggrecan must integrate a certain amount of CS to maintain its normal function. Therefore, a lack of CS is thought to be the main cause of dehydration during IDD progression [19]. However, the regulatory mechanism of the biosynthesis process of CS in IDD progression is still poorly understood. Inflammatory cytokines play key roles in the pathophysiological process of IDD [20–22]. Interleukin-1β (IL-1β) and tumor necrosis factor-α (TNF-α) are the primary pro-inflammatory cytokines that are up-regulated in degenerated discs. They are implicated to increase the activities of MMPs, ADAMTs, and decrease synthesis of collagen type II, aggrecan and CS in animal NP cells [20, 21, 23]. However, there is no evidence regarding whether such cytokines can directly affect the anabolism of CS in human NP cells.

Here, we set out to find the underlying mechanism of impaired CS biosynthesis during IDD. By examine the expression of CS glycosyltransferases in tissue samples, we found that the expression of CHSY-1, -2, -3 was significantly lowered in more degenerated NP tissues. Furthermore, *in vitro* analysis revealed that the gene expressions of CHSY-1, -2, -3 can be affected by inflammatory cytokines and is greatly influenced in the protein level, but not the mRNA level. Through mechanism study, we showed that IL-1β and TNF-α stimulated microRNA-194, -515 expression can significantly reduce CHSY expression and thus lower CS biosynthesis. Our study here provided new knowledge about the dysregulation mechanism of CHSY during IDD, and established a cytokine-microRNA regulation network that modulated CS synthesis in nucleus pulposus.

## RESULTS

### CS content decreased significantly in degenerated nucleus pulposus tissues

**Figure 1:**
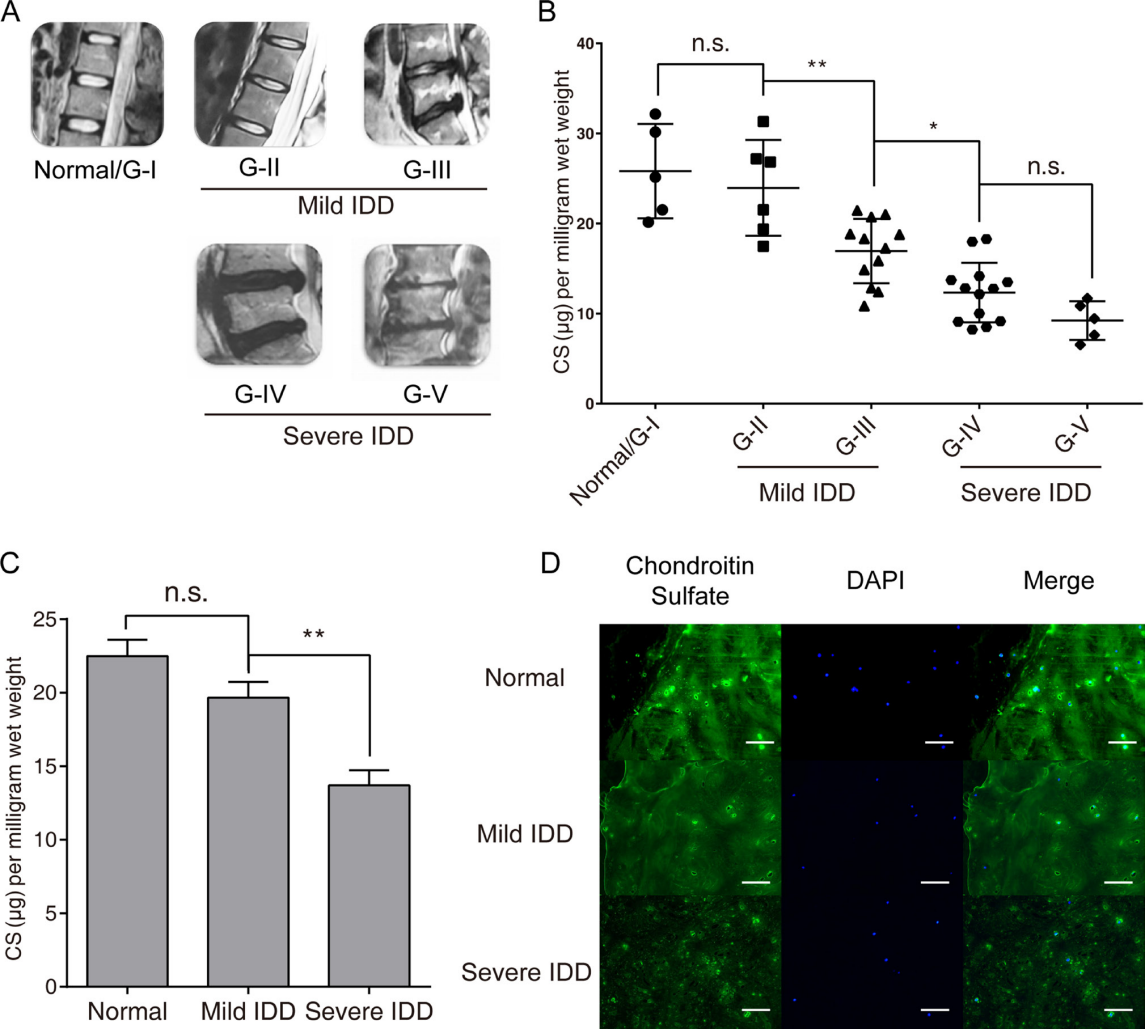
CS concentration reduced significantly in degenerated human intervertebral disc tissues (**A**) Disc tissues from patients were collected and classified using the modified Pfirrmann grading system, and samples of grade II/III (*n* = 18) were assigned to the mild IDD group, samples of grade IV/V (*n* = 18) were allocated to the severe IDD group, samples of grade I (*n* = 5) were allocated to the normal group. (**B**–**C**) DMMB assay shows that the CS concentration in NP samples from each sample divided by Pfirrmann grade (**B**) and the mean CS concentration between mild IDD, severe IDD and normal NP samples (C). The results are normalized to the tissue wet weight, and data are shown as mean ± SD. *p < 0.05; **p < 0.01. (**D**) Representative Immunofluorescence microscopy images showing the CS staining between normal disc, mild IDD NP and severe IDD NP samples, see also Supplementary Figure 1A. Bars represents 200 μm.

To gain initial information about CS synthesis in IDD, we first evaluated the overall CS content in NP samples of different degeneration grade. The degree of IDD was estimated by MRI imaging according to the modified Pfirrmann grading system (Figure 1A). Of the 41 samples analyzed, 5 NP samples of grade I were categorized as normal group; 18 NP samples of grade II and III were categorized as the mild IDD group; the other 18 specimens of grade IV and V were categorized as the severe IDD group. The CS concentration in NP tissues was presented relative to the wet weight of the tissues. We found that the amount of CS gradually decreases as degeneration grade increases (Figure 1B), while mean CS concentration was significantly decreased in severe IDD group compared with mild IDD group (Figure 1C). Immunofluorescence microscopy of the human NP sections showed that CS exhibited a more uniform and compact distribution in the normal and mild IDD group compared with the severe IDD group (Figure 1D), while higher mean fluorescence intensity was also detected in the mild IDD group (Figure 1D, Supplementary Figure 1A). These findings suggested that the CS content is indeed in reverse correlation with the degree of degeneration.

### Assessing the expression of CS glycosyltransferases in degenerated nucleus pulposus

**Figure 2:**
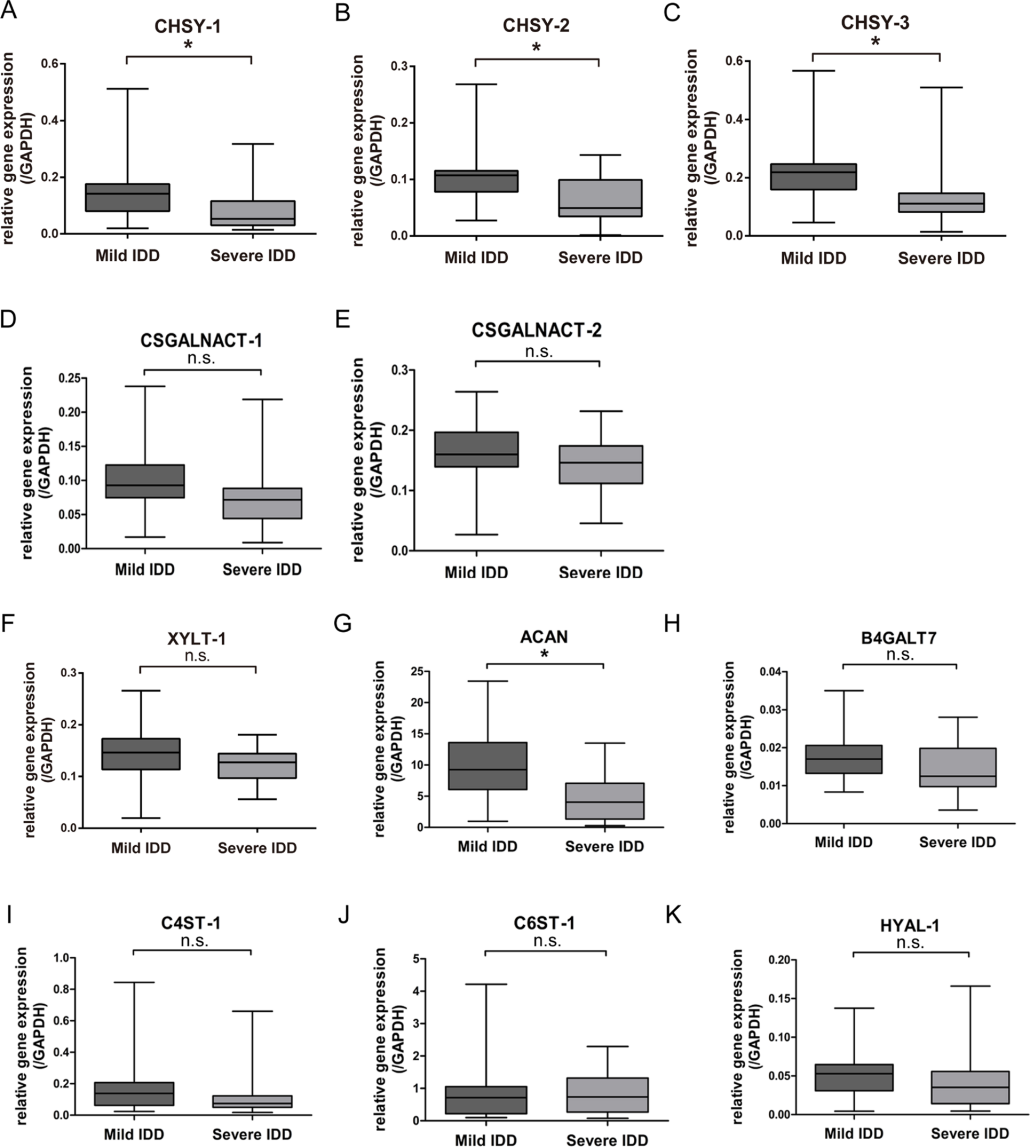
Assessing the expression of CS related enzymes in mild and severe IDD NP samples (**A**–**E**) Real-time PCR analysis assessing the mRNA levels of CHSY-1 (A) CHSY-2 (B) CHSY-3 (C) CSGALNACT-1 (D) CSGALNACT-2 (E) XYLT-1 (**F**) and ACAN (**G**) B4GALT7 (**H**) C4ST-1 (**I**) C6ST-1 (**J**) and HYAL-1 (**K**) between mild and severe IDD NP samples. *n* = 18 for each group, *represents *p* < 0.05, n.s. represents no significance. The data were normalized to GAPDH.

In order to find clue for the loss of CS content during IDD progression, we analyzed the expression of CS glycosyltransferases between mild and severe IDD NP tissues. We first performed real-time PCR (qPCR) to measure the mRNA expression of CHSY-1, -2, and -3, and of CSGALNACT-1 and -2 in mild and severe degenerated tissues. The results showed that the mRNA level of CHSY-1, -2, and -3 were significantly decreased in severe cases compared to mild cases (Figure 2A–2C), while the mRNA level of *CSGALNACT-1* and *-2* did not show significant changes between groups (Figure 2D–2E).

To confirm the role of these CS glycosyltransferases in causing CS biosynthesis defection in degenerated NP, we tested the expression of other functional enzymes and genes that cooperate to synthesize CS. The mRNA levels of XYLT-1, ACAN, B4GALT7, C4ST-1, C6ST-1, and HYAL-1 were measured using qPCR. Results showed that only the mRNA level of ACAN was significantly decreased in severe IDD compared to mild IDD group (Figure 2F–2K), while differences in *XYLT-1*, *B4GALT7*, *C4ST-1*, *C6ST-1*, and *HYAL-1* between the mild and severe IDD group did not reach statistical significance. Taken together, we found that among the enzymes that synthesize CS, the mRNA level of glycosyltransferases CHSY-1, -2, and -3 down-regulated significantly in severe IDD NP samples, indicating the vital roles of these genes in degeneration.

### CHSYs expression significantly decreased in more degenerated nucleus pulposus tissues

**Figure 3:**
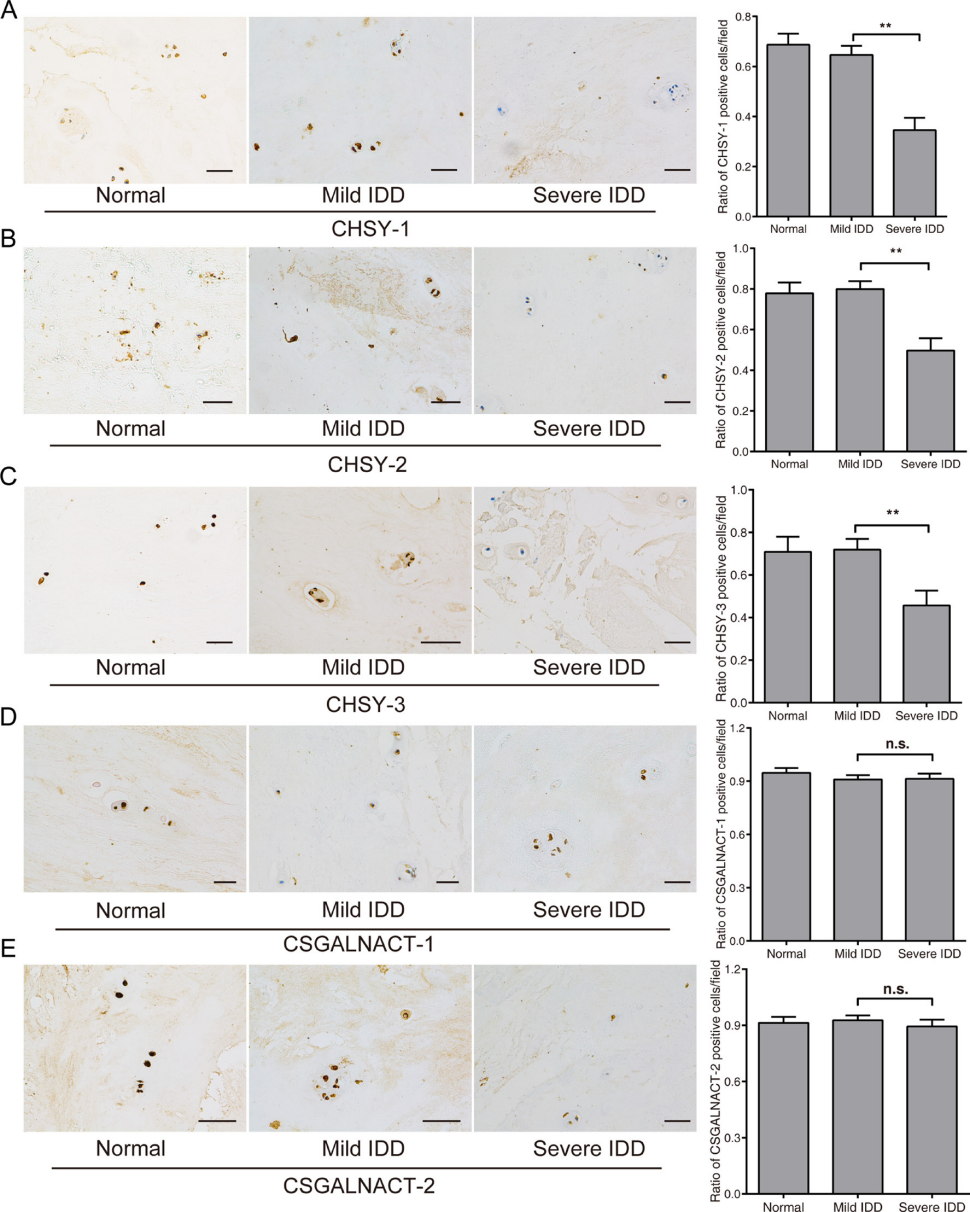
Immunohistochemistry analysis of CS glycosyltransferases in mild and severe NP tissues Immunohistochemistry analysis was performed to measure the protein level of five CS glycosyltransferases, and representative images of the NP cells stained for CHSY-1 (**A**), CHSY-2 (**B**), CHSY-3 (**C**), CSGALNACT-1 (**D**), CSGALNACT-2 (**E**) were shown in the left panels. *n* = 5 for each group, bars represents 200 μm. Quantifications of positive cells in the IHC analysis were shown in the right panels. Data are shown as mean ± SD. *p < 0.05; **p < 0.01.

To gain more evidence of dysregulated CS glycosyltransferases in disc degeneration, we performed immunohistochemical analysis (IHC) of the CS glycosyltransferases in normal, mild and severe IDD NP specimens. Expression of CHSY-1, -2 and -3 showed a similar pattern, with obvious staining occurred in normal and mild IDD group, while no staining of CS glycosyltransferases were found in severe IDD groups (Figure 3A–3C). However, the expression of CSGALNACT-1 and -2 did not show significant changes (Figure 3D–3E), which is consistent with previous results of qPCR. To further quantify the results, we counted the positively stained cells for each samples and calculated the CS glycosyltransferases positive cell ratio. Results showed that the ratio of CHSY-1, -2 and -3 were significantly lower in severe IDD compared with that in mild IDD NP tissues (Figure 3A–3C), with the significance much higher than qPCR results (Figure 2A–2C). The ratio of CSGALNACT-1 and -2 positive cells did not reach statistical significance (Figure 3D–3E), which is consistent with previous findings. Taken together, our results showed that the expression of CHSY-1, -2 and -3 in severe degenerated NP is indeed lower than mild group.

### Inflammatory cytokines affect CS glycosyltransferases expression *in vitro*

**Figure 4:**
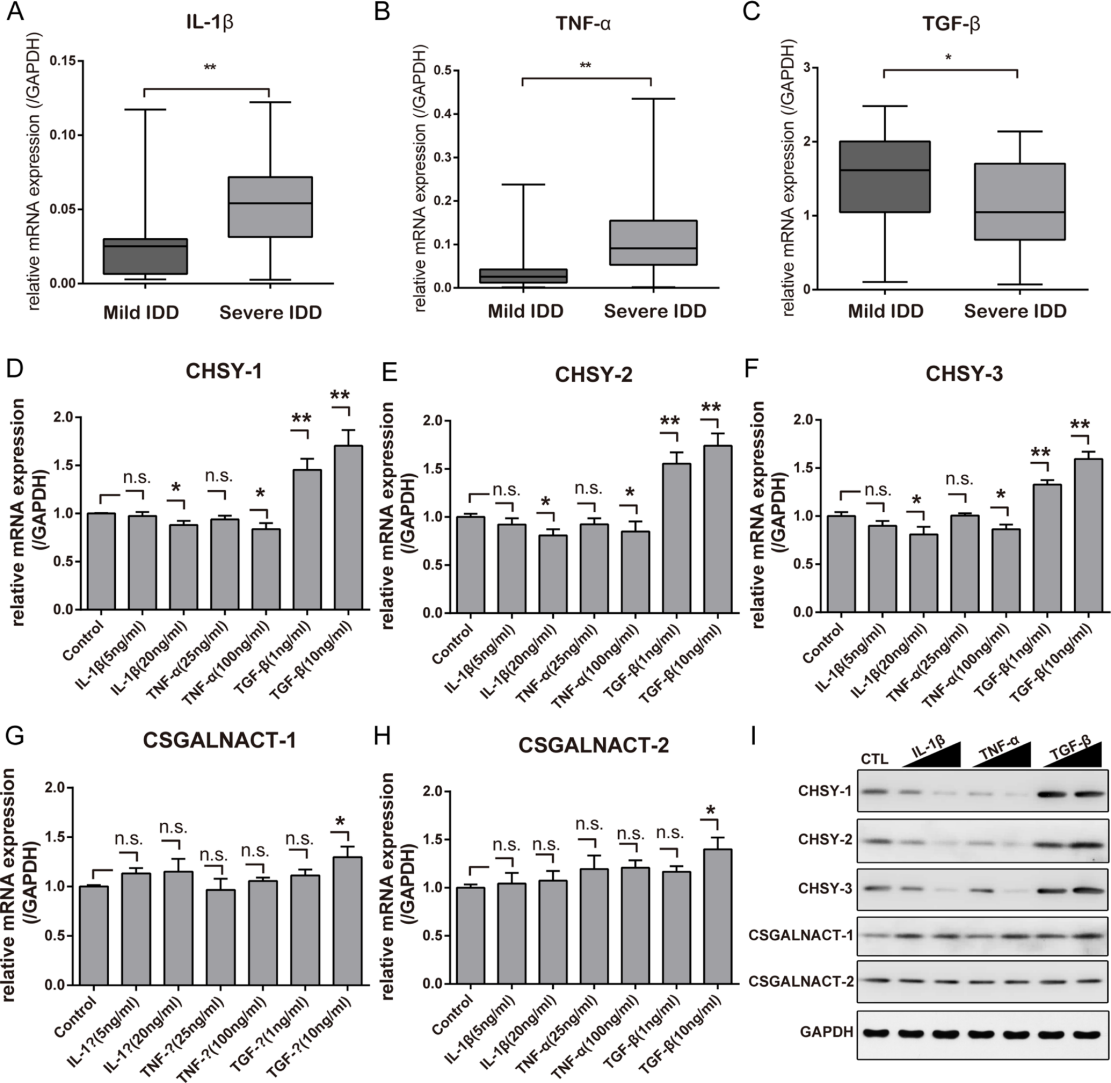
Inflammatory cytokines affect CHSY expressions *in vitro* Real-time PCR analysis showing the mRNA level of IL-1β (**A**), TNF-α (**B**) and TGF-β (**C**) between mild and severe IDD NP tissues. *p < 0.05; **p < 0.01, *n* = 18 for each group. The mRNA level of CHSY-1 (**D**), CHSY-2 (**E**), CHSY-3 (**F**), CSGALNACT-1 (**G**), and CSGALNACT-2 (**H**) under different inflammatory cytokine stimulation were tested using Real-time PCR. Data are shown as mean ± SD. *p < 0.05; **p < 0.01, *n* = 3, n.s. represents no significance. Western blot analysis showing the protein level of CHSY-1, CHSY-2, CHSY-3, CSGALNACT-1 and CSGALNACT-2 (**I**). The solid triangle represents increased doses of inflammatory cytokine.

It is known that inflammatory cytokines involved in IDD progression. Here, we first confirmed the expression of the pro-inflammatory cytokines IL-1β and TNF-α and the anti-inflammatory cytokine TGF-β in tissue samples using qPCR. The results showed that the expression of IL-1β and TNF-α were significantly up-regulated in the severe IDD group compared with those in the mild IDD group, while TGF-β showed the opposite result (Figure 4A–4C).

To determine the effects of the IDD-related inflammatory cytokines on expression of CS glycosyltransferases, NP cells from IDD patients were isolated and treated with IL-1β, TNF-α or TGF-β for 48 h. After treatment, the gene expression of five CS glycosyltransferases was determined using qPCR. Results showed that both IL-1β and TNF-α stimulation did not led to a significant dose dependent down-regulation of the mRNA level of all CS glycosyltransferases (Figure 4D–4H), significance only occurred in CHSY-1, -2 and -3 mRNA level under high dosage of IL-1β and TNF-α stimulation (Figure 4D–4F). However, TGF-β treatment significantly upregulated the mRNA level of three CHSYs (Figure 4D–4F), with less significant effects to CSGALNACT-1 and -2 (Figure 4G–4H). To further confirm this phenomenon, we performed Western Blot analysis. Different from the qPCR results, the Western Blot analysis revealed significant CHSYs downregulation under IL-1β and TNF-α stimulation, while CSGALNACT-1 and -2 showed no significant changes (Figure 4I). Taken together, we showed that inflammatory cytokine IL-1β and TNF-α significantly downregulate the protein level of CHSY-1, -2 and -3, but not the mRNA level *in vitro*. These results are in accordance with previous findings in tissue samples, which indicate post-transcriptional regulations exist in IL-1β and TNF-α modulation of CHSYs expression in NP cells.

### IL-1β and TNF-α stimulated miR-194 and -515 target CHSYs in human NP cells

**Figure 5:**
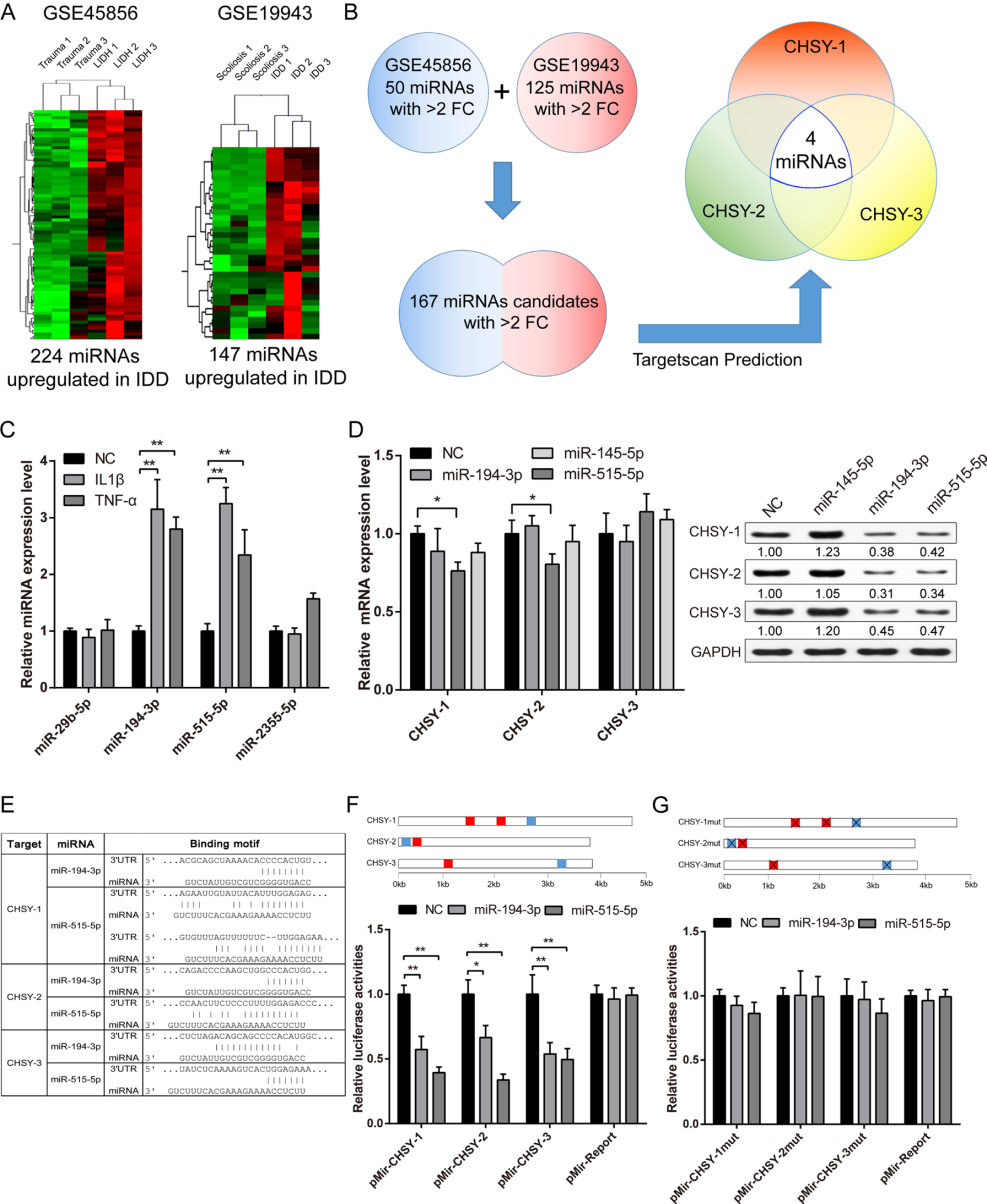
Screening and validation of CHSY targeted microRNAs (**A**) Representative images of hierarchical cluster showing the differentially expressed miRNAs among non-degenerated and degenerated NP tissues. Red represents highly expressed miRNA, while green represents reduced expression. Trauma and scoliosis samples were NP tissues obtained from these patients with no sign of disc degeneration, which represents normal NP, while LIDH and IDD represents degenerated NP tissues. (**B**) A scheme of computational prediction of CHSYs targeted miRNAs. Only miRNAs that target all three CHSYs were selected for further analysis. (**C**) Real-time PCR analysis showing the expression of candidate miRNAs under IL-1β and TNF-α stimulation. Data are shown as mean ± SD. **p < 0.01, *n* = 3, all expression were normalized to U6 expression as internal reference. (**D**) Real-time PCR analysis showing the expression of CHSYs under miRNA transfections. Data are shown as mean ± SD. *p < 0.05, *n* = 3, data were normalized to GAPDH, a scramble miRNA mimic control that target none of the CHSYs was used as negative control, here miR-145 was also served as a negative control. The right panel shows the relative protein level of CHSYs, the quantification of gray scale were shown under the blot, *n* = 3. (**E**) A list of target sites of miR-194 and -515 in CHSYs mRNA. (**F**–**G**) Dual luciferase reporter assay of wildtype (F) and mutated (G) CHSY 3′UTR reporters under miR-194 and -515 overexpression. The blue squares represent miR-194 targeted sites, while red square represents miR-515 targeted sites. Data are shown as mean ± SD. *p < 0.05, **p < 0.01, *n* = 6, data were normalized to *Renilla* luciferase activities.

In order to evaluate the involvement of microRNA (miRNA), an important post-transcriptional regulator, in CHSY regulation, we first take advantage of IDD high through-put microRNA profiling data from GEO (Gene Expression Omnibus) datasets. We chose two human IDD microRNA profiling data, GSE45856 and GSE 19943, to screen for further analysis (Figure 5A). To eliminate patient individual differences, we combined the two datasets and use stringent criteria (microRNAs that have more than 2 fold changes between groups are considered as candidates) to screen for possible candidates. Altogether 167 miRNAs passed the criteria, and we further examined their relation to CHSYs by using Targetscan prediction algorithm (http://www.targetscan.org, Figure 5B). By miRNA target prediction, we found 4 miRNAs (miR-29b, -194, -515, -2355) that could target CHSY-1, -2 and -3 at the same time, which we select for further analysis (Figure 5B).

We first tested whether these candidate miRNAs can be stimulated by inflammatory cytokine IL-1β and TNF-α. Using qPCR, we found that miR-194 and miR-515 were highly upregulated after cytokine stimulation (Figure 5C), indicating miR-194 and -515 may take vital roles in IL-1β and TNF-α modulated CHSYs expression. By directly overexpressing miRNA mimics, we found that the mRNA level of CHSYs did not change significantly except for miR-194 overexpression group (Figure 5D). However, the western results showed significant downregulation in both miR-194 and -515 overexpression groups, which is consistent with that of IL-1β and TNF-α treatment (Figure 5D). We also tested their effect on CSHALNACT-1 and -2. As expected, no significant changes were observed in both mRNA and protein level (Supplementary Figure 1B).

To further validate their functional mechanism, we constructed wild type and target site mutated luciferase reporters (Figure 5E). We performed dual luciferase reporter assay to determine the miRNAs' effect on CHSY 3′UTRs (Untranslated regions). As expected, results showed that miR-194 and -515 significantly decreased the luciferase activities of CHSY-1, -2 and -3 (Figure 5F), while no significant changes were observed using mutant reporters (Figure 5G). Taken together, we found that IDD upregulated and IL-1β/TNF-α stimulated miR-194 and -515 could affect the protein translation of CHSY-1, -2 and -3 through direct binding to their 3′UTR.

### MiR-194 and -515 are responsible for IL-1β and TNF-α modulated CHSYs expression in degenerated NP

**Figure 6:**
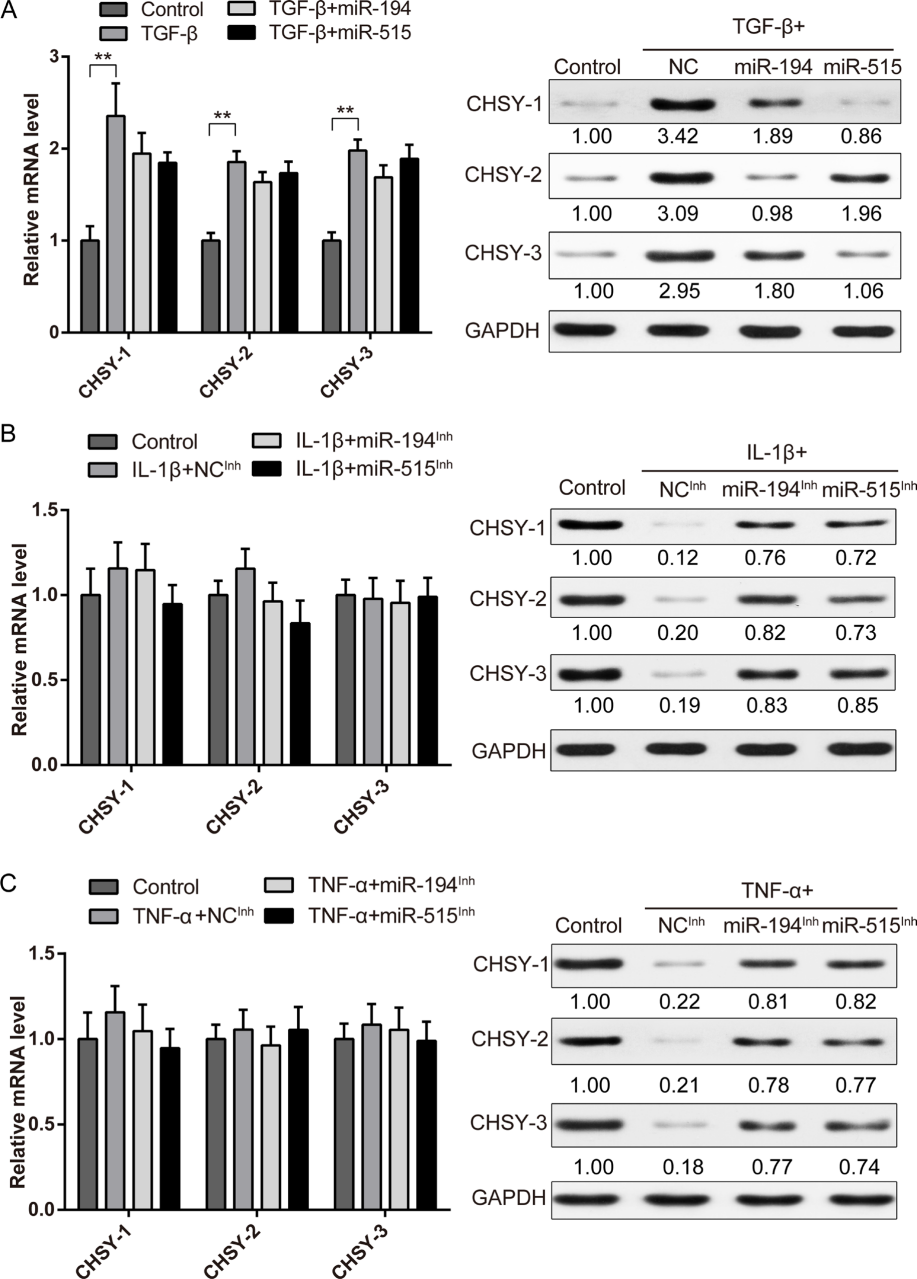
MiR-194 and miR-515 is essential for inflammatory cytokine regulated CHSY expressions Real-time PCR and Western blot analysis showing the expression level of CHSY-1, -2 and -3 under TGF-β (**A**), IL-1β (**B**), TNF-α (**C**) treatment combined with miR-194, -515 or control miRNA mimic overexpression (A) or inhibition (B-C). Data are shown as mean ± SD. **p < 0.01, *n* = 3, data were normalized to GAPDH. The right panel shows the relative protein level of CHSYs, the quantification of gray scale were shown under the blot, *n* = 3.

Now that we know miR-194 and -515 could affect CHSYs expression directly, however, their roles in IL-1β and TNF-α modulated CHSYs expression is not known. Here we first confirmed the regulation ability of miR-194 and -515 in TGF-β stimulated NP cells. We found that miR-194 and -515 can indeed reduce the upregulated CHSYs expression at protein level, while showed no significance in mRNA level (Figure 6A). Next, we use miRNA antisense inhibitors to eliminate the function of endogenous miR-194 and -515 to test their roles in IL-1β and TNF-α stimulation. Results showed that after IL-1β and TNF-α stimulation, the protein level of CHSYs decreased significantly. With the addition of miR-194 and -515 inhibitors, protein expression of CHSYs upregulated significantly, while mRNA level remain unchanged (Figure 6B–6C). These results indicated that inhibition of miR-194 and -515 during IL-1β and TNF-α stimulation can indeed rescue the downregulated expression of CHSYs, which indicated a vital role of miR-194 and -515 in the IL-1β and TNF-α modulated CHSYs expression.

**Figure 7:**
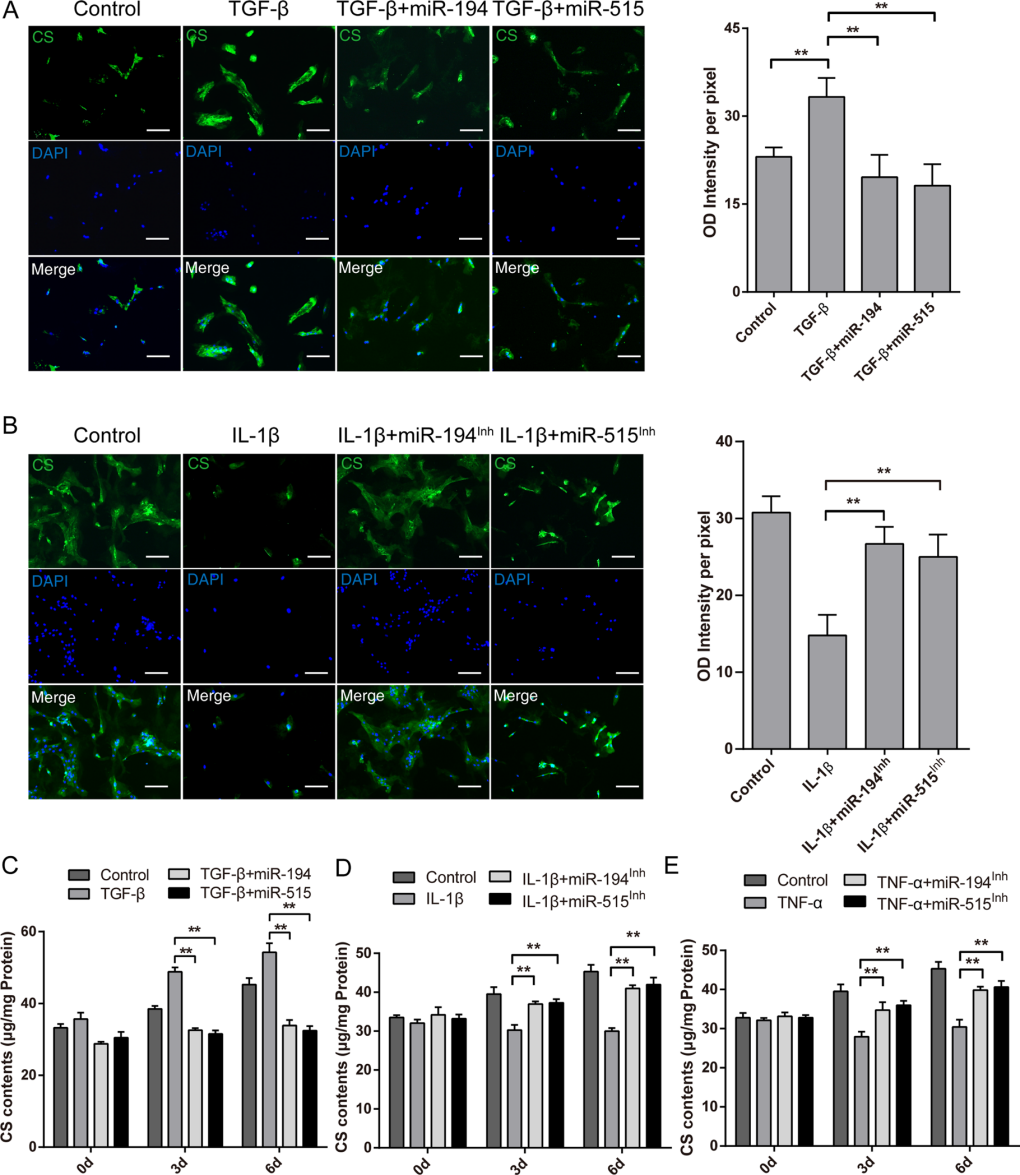
Effects of miR-194, -515 on inflammatory cytokines modulated chondroitin sulfate content in human NP cells (**A**) Immunofluorescence images of CS in NP cells treated with 10 ng/ml TGF-β and miR-194 or miR-515 or scramble miRNA mimic control for 72 h. (**B**) Immunofluorescence images of CS in NP cells treated with 20 ng/ml IL-1β and miR-194 or miR-515 inhibitors or scramble control inhibitor for 72 h. The quantifications of relative intensity were shown in the right panel. Data are shown as mean ± SD. **p < 0.01, *n* = 5, bars represents 50 μm. Cells and culture medium were collected to perform the DMMB assay at 3, 6, or 9 days after TGF-β and miRNA overexpression treatment (**C**), IL-1β and miRNA inhibition treatment (**D**), or TNF-α and miRNA inhibition treatment (**E**). Data are shown as mean ± SD. **p < 0.01, *n* = 3.

Next we examined whether CS content is affected by miR-194 and -515. We first performed CS Immunofluorescence assay to assess their level. Consistent with previous findings on CHSYs modulation, the signal of CS Immunofluorescence is significantly lowered after miR-194 and -515 treatment compared with the TGF-β treatment group (Figure 7A). On the other hand, inhibition of miR-194 and -515 significantly rescued the diminished CS Immunofluorescence caused by IL-1β and TNF-α stimulation (Figure 7B, Supplementary Figure 1C). Moreover, we performed DMMB assay to assay the CS concentration in these groups. To our expect, miR-194 and -515 treatment significantly halted the upregulation of mean cellular CS concentration stimulated by TGF-β (Figure 7C), while their inhibition increased the mean cellular CS concentration compared with IL-1β and TNF-α treatment (Figure 7D–7E). Till now, we found that miR-194 and -515 can indeed affect the CS content synthesized by NP cells, and the mechanism of which is related to IL-1β and TNF-α modulated CHSYs expression.

## DISCUSSION

Loss of water content is the key pathogenic process of IDD, and early studies provided evidence that CS plays an important role in the water binding capacity of the intervertebral disc, especially in the NP tissue [9, 24]. Nishiyama [17] found that the CS concentration and chain length are both reduced in IDD, indicating that changes in the CS content are associated with IDD progression. Other recent studies revealed that inflammatory cytokines regulate the metabolism of the ECM, leading to increased catabolic activity and decreased anabolic ability [20–22]. However, the underlying mechanism and the relationship between these cytokines and CS metabolism are still poorly understood. Better understanding of these pathological processes might shed light on novel therapeutic options to relieve pain and inhibit IDD progression. Consistent with previous studies [17, 18], we here show that the CS concentration is lower in human NP samples with severe IDD than in mild IDD. In addition, we show here that the expression of CS glycosyltransferases is regulated by IDD-related inflammatory cytokines through miRNA mechanism. To our knowledge, this is the first study to report such regulation pathway combining CS content, CS glycosyltransferases, inflammatory cytokines and miRNAs in human intervertebral disc.

Ishimaru and colleagues [25] found that the CS concentration in OA cartilage was lower compared with that in normal cartilage, and this was correlated with the impaired expression of CS biosynthesis enzymes. Since NP cells have a similar morphology and phenotype to chondrocytes, we hypothesized and showed that both the gene and protein expressions of CHSY-1, -2 and -3, which are responsible for the biosynthesis of CS, are reduced with the degree of degeneration. Regarding the catabolic aspect, the catabolism of CS is currently poorly understood and the CS catabolic enzymes identified at present include HYAL-1 and -4 [26, 27]. However, HYAL-4 has only been shown to be expressed in placenta, skeletal muscle and testis [27], and we found no significant difference in expression of HYAL-1 between mild and severe IDD.

The biosynthesis of CS chains starts with the creation of a tetrasaccharide unit (CS linkage region) and is elongated by polymerization of repeating disaccharide units (CS backbone region) [28]. CHSY-1 presents the highest glycosyltransferase activity and mainly functions in the elongation of CS chains [12, 25]. Knockdown of CHSY-1 by small interfering RNA (siRNA) causes a decrease in the number and molecular weight of CS chains, and CHSY-1 null mice exhibit chondrodysplasia and defects in chondroitin sulfation balance [29, 30]. Similar to CHSY-1, CHSY-2 and -3 also participates in CS backbone elongation [28]. Overexpression of CHSY-3 enhances the amount of CS, while knockdown of CHSY-3 results in a reduction in the amount of CS [28]. We found that the expression of CHSY-1, -2 and -3 were all down-regulated in severe IDD, suggesting that the reduced expression of these CS glycosyltransferases might contribute to the loss of CS content in IDD by impairing CS biosynthesis. However, CSGALNACT-1 and 2 are also critical enzymes involved in CS chain initiation and elongation [15, 16], but we failed to detect any significant differences in expression at either the gene or the protein level, indicating a less significant role of them in IDD. More interestingly, we found that the reduced mRNA level of CHSYs in severe IDD is less significant than their protein level, while similar phenomenon was found during *in vitro* study.

Such regulation pattern is very similar to that of miRNA regulation, with classic miRNA targeting events often take part in the 3′ untranslated region (3′UTR) of the target mRNA, resulted in translational repression rather than mRNA degradation. Traditionally, microRNA form the RISC complex with several proteins like Ago to conduct its gene translational repression function or the so called post-transcriptional regulation. During this process, microRNA bind to the 3′-UTR regions of the target mRNA. However, if the target and the microRNA form an imperfect binding, the target mRNA is likely to be translational repressed rather than degradation of the mRNA [31]. In our study, since inflammatory cytokines like IL-1β and TNF-α could stimulate expression changes of a variety of miRNAs, we hypothesized that miRNAs are major regulators that connect inflammatory cytokines with CS glycosyltransferases. We found that miR-194 decreases the protein level of CHSY-1, -2, -3, but not the mRNA level. To further verify that the phenomenon is caused by an imperfect binding of miR-194 to CHSY-1, -2, -3 3′-UTR region, we performed dual-luciferase reporter assay and confirmed the mechanism. Such cytokine-microRNA-proteoglycan regulatory axis is also noted in *Caenorhabditis Elegans*, in which miR-79, an ortholog of mammalian miR-9 controls the expression of chondroitin synthase and disrupts neuronal migration [32]. Another study showed that axonal microRNAs regulate local proteoglycan composition to mediate axonal growth [33]. These studies together emphasized that microRNA mediated extracellular matrix remodeling is crucial to many biological processes. However, in our study, we proved that miR-194 and -515 is the key mediator between inflammatory cytokines and CS synthesis which demonstrated a new regulatory circuitry in IDD pathogenesis, and also gave evidence that microRNAs are also important regulators in intervertebral disc degeneration.

## MATERIALS AND METHODS

### Collection and grading of human tissue samples

Informed consent was provided by the patients or their relatives to obtain human intervertebral tissue at surgery. The experiment was authorized by the ethics committee of Second Military Medical University. Thirty-six nucleus pulposus samples with different Pfirrmann grade (II-V) of disc degeneration (*n* = 36; age 25 to 78 years, mean 47 years) were obtained from patients (Supplementary Table 1) who underwent disc resection surgery or spinal fusion to relieve CLBP. Five samples of Pfirrmann grade I (normal, *n* = 5; age 35 to 58 years, mean 43 years) disc samples were obtained from lumbar trauma patients who underwent spinal fusion. Patients diagnosed with classical sciatica were excluded from the study. MRI T-2 weighted images were collected and the modified Pfirrmann grading system [34] was used to evaluate the degree of IDD. In this study, samples of grade-I (G-I) were classified as the normal group, samples of grade-II (G-II) and grade-III (G-III) were classified as the mild IDD group, grade-IV (G-IV) and grade-V (G-V) samples were classified as the severe IDD group. Due to the availability and uncertainty of tissue sample collection, the sample number in each groups are not evenly distributed. Nevertheless, we included all the collected samples to be analyzed in the study.

### Nucleus pulposus cell extraction

For cell extraction, NP tissue specimens were washed twice with PBS, then minced and digested with 2 U/mL protease in DMEM/F12 (Gibco, Grand island NY, USA) for 30 minutes at 37°C. NP cells were released from the tissues by treating with 0.25 mg/mL type II collagenase (Gibco, Cat. No. 17101-015) for 4 hours at 37°C. The resulting cell suspension was transferred into a 40 μm cell strainer (BD Falcon, Becton Dickinson, Franklin Lakes, NJ, USA) and centrifuged at 800 g for 5 minutes. The NP cells were resuspended in DMEM/F12 containing 10% FBS (Gibco), 100 U/mL penicillin, 100 μg/mL streptomycin, and 1% L-glutamine. Cells were incubated at 37°C in 5 % CO_2_ and the medium was changed every 3 days. Cells at passage two and on were used for all subsequent experimental procedures.

### Nucleus pulposus cell culture and treatment with inflammatory cytokines

When the NP cell culture became confluent, the cells were trypsinized with 0.25% trypsin/ethylenediaminetetraacetic (Gibco) and seeded into six-well plates at 150,000 cells/well in the same medium. When the cells in the plates reached confluence, the cultures were treated with the following cytokines: 5/25 ng/mL of IL-1β, 10/100 ng/mL of TNF-α or 1/10 ng/mL of TGF-β (all from Peprotech, Rocky Hill, NJ, USA) in medium for 48 h at 37°C, in 5% CO_2_.

### RNA extraction and reverse transcription

RNA was extracted from human nucleus pulposus samples using Trizol (Invitrogen, Carlsbad, CA) according to the manufacturer's instructions. Concentration of total RNA was measured at 260 nm with a spectrophotometer (DU-800; Beckman Coulter, Brea, CA). First strand complementary DNA (cDNA) synthesis was performed with 500 ng of total RNA in a 10 μL final volume containing 2 μL Primer Script RT Master Mix (Takara, RR036A, Japan) and 8μLof RNase-free dH_2_O and total RNA. The reverse transcription procedure was carried out according to the manufacturer's instructions.

### Quantitative real-time polymerase chain reaction

Real-time PCR for glyceraldehyde-3-phosphate dehydrogenase (GAPDH), CHSY-1, 2, and 3, CHPF, CSGALNACT-1 and 2, aggrecan (ACAN), xylosylprotein beta 1,4-galactosyltransferase, polypeptide-7 (B4GALT-7), C4ST-1, C6ST-1, xylosyltransferase-1 (XYLT-1), hyaluronidase-1 (HYAL-1), IL-1β, TNF-α and TGF-β was performed by using SYBR premix Ex Taq™(Takara Bio Inc., Shiga, Japan) with a Step One Plus real-time PCR system (Applied Biosystems, Foster City, CA), according to the manufacturer's instructions. GAPDH was used to normalize the gene expression of mRNAs. The relative amount of transcripts was calculated according to the comparative Ct method. All of the primers were synthesized by Invitrogen (Supplementary Table 2).

### Immunohistochemical procedure

Immunohistochemistry was conducted to localize CHSY-1, 2, 3, CSGALNACT-1, and 2 in 5 NP samples of Pfirrmann grade-I, 6 NP samples of Pfirrmann grade-II, 12 NP samples of grade-III, 13 NP samples of grade-IV, and 5 NP samples of grade-V. The general immunohistochemistry protocol was as described elsewhere [35]. Briefly, antigen retrieval was performed using trypsin for 30 min at 37°C, and the sections were blocked with 1% bovine serum albumin for 15 min at room temperature. Next, the sections were incubated at 4°C overnight with the primary antibodies as follows: rabbit polyclonal antibody against CHSY-1 (1:100 dilution, Proteintech Group Inc., Chicago, IL, USA; catalog no. 14420-1-AP), rabbit polyclonal antibody against CHSY-2 (1:100 dilution, Bioss Antibodies, Woburn, MA, USA; catalog no. bs-12939R), goat polyclonal antibody against CHSY-3 (1:100 dilution, Santa Cruz Biotechnology, Santa Cruz, CA, USA; catalog no. sc-50551), rabbit polyclonal antibody against CSGALNACT-1 (1:100 dilution, Abgent, San Diego, CA; catalog no. AP4922c), and rabbit polyclonal antibody against CSGALNACT-2 (1:100 dilution, GeneTex Inc., Irvine, CA; catalog no. GTX81034). After washing, the sections were incubated with the secondary antibody peroxidase-conjugated Affinipure Goat Anti-Rabbit IgG (1:1000 dilution, Proteintech; catalog no. SA00001-2) or with the ImmunoCruz™ goat LSAB Staining System (Santa Cruz Biotechnology; catalog no. sc-2053), then, the sections were counterstained with hematoxylin.

### DMMB assay

The DMMB (1,9-dimethylmethylene blue) assay was performed using a Blyscan™ sGAG assay kit (Biocolor, B1000, Biocolor, Carrickfergus, UK) according to the manufacturer's instructions. Briefly, harvested tissues and NP cells were washed with PBS, and digested with 1mL papain extraction reagent for 24 h at 65°C. CS contents were determined by reaction with DMMB, and staining was quantified by measuring absorbance at 656 nm. Chondroitin-4-sulfate was used as the standard.

### Immunofluorescence microscopy

Human NP cells were plated into a 24-well plate (10,000 cells/well) and treated with IL-1β, TNF-α or TGF-β for 72 h. At the end of the incubation period the cells were fixed with 4% paraformaldehyde and permeabilized with 0.3% Triton X-100 in PBS for 10 min. Next, the cells were blocked with 5% bovine serum albumin in PBS and incubated with a mouse monoclonal antibody against chondroitin sulfate (1:200 dilution) (Sigma-Aldrich, St Louis, MO, USA; catalog no. C8035) at 4°C overnight. The cells were washed with PBS and incubated with a goat anti-mouse IgG-FITC secondary antibody (1:128 dilution) (Sigma-Aldrich; catalog no. F5262) for 1 h at room temperature. Lastly, samples were counter stained with DAPI (4′, 6-diamidino-2-phenylindole, 1:1000 dilution, Beyotime, Shanghai, China) for 5min at room temperature. Samples were viewed under a fluorescence microscope (ZEISS, Axio Imager A2). The same immunofluorescence protocol was also used for the NP sections to determine the localization of CS in different NP tissues. Quantification was performed using ImageJ (Version 1.5.1a, https://imagej.nih.gov/) by analyzing six random field of each group and the mean intensity per pixel was calculated. In brief, the Integrated Density of each immunofluorescence images were calculated as the intensity of fluorescence. Then the background fluorescence readings were corrected by calculating the Integrated Density of background compared to positive stained cells. The Corrected Total Cell Fluorescence (CTCF) were final calculated and compared by the formula: Integrated Density – (Area of selected cell X Mean fluorescence of background readings).

### Plasmid construction, gene transfection, and Luciferase reporter assay

Reporter construction was performed by synthesizing wild type or mutated 3′UTR and subcloned into pMir-report vector (Promega, WA, USA), which all procedure were done by Obio Technology Corp (Shanghai, China). NP cells were plated in 96-well plates and transfected using Lipofectamine 2000 (Invitrogen) with 50 ng pMir-report vector (carrying firefly luciferase) with indicated 3′UTR of wild type or mutated CHSY-1, -2 or -3. Empty vector was served as a control, and a PRL-TK vector (carrying Renilla luciferase) was served as internal control (Promega). Besides, the indicated microRNAs or scramble control were transfected simultaneously (20 pmol per well). 24 hours after transfection, cells were then fixed and measured both the firefly and Renilla luciferase activities according to the manufacturer's instructions.

### Computational analysis

The normalized microRNA profiling data were searched and downloaded from Gene Expression Omnibus (GEO) repository. The miRNA ID of each data (GSE45856 and GSE19943) were updated with miRbase v21 and thus can be integrated into a single dataset. Quintile normalization method was applied to scale each dataset to a uniform expression level. Unsupervised clustering using average linkage and median centering were sub sequentially performed. MicroRNA target prediction were performed using Targetscan (http://www.targetscan.org). The candidate microRNAs were submitted to the site to search for potential binding sites in the 3′ untranslated region of CHSY-1, 2, 3.

### Statistical analysis

Data are presented as mean ± standard deviation for at least three independent experiments. GraphPad Prism 6.0 software (GraphPad Software, Inc., San Diego, CA, USA) was used for statistical analysis. The normality of the data was tested using the D'Agostino-Pearson omnibus normality test. The data of relative gene expression (CHSY-1, 2, 3, CSGALNACT-1, 2, ACAN, XYLT-1, B4GALT-7, C4ST-1, C6ST-1, HYAL-1, IL-1β, TNF-α and TGF-β) and the quantification study of immunopositive NP cells did not pass the normality test; therefore these data were analyzed by applying the Mann-Whitney *U* test. The remaining data passed the normality test, and Student's *t*-test or analysis of variance (ANOVA) followed by Tukey's *t*-test were performed for comparison of two groups or multiple groups, respectively. *P* < 0.05 was considered significant.

## SUPPLEMENTARY MATERIALS FIGURE AND TABLES



## Abbreviations

CS: Chondroitin sulfate
NP: nucleus pulposus
IDD: Intervertebral disc degeneration
CLBP: Chronic low back pain
ECM: extracellular matrix
AF: annulus fibrosus
MMPs: metalloproteinases
ADAMTs: a disintegrin and metalloproteinase with thrombospondin motifs
GAG: glycosaminoglycan
GalNAc: N-acetylgalactosamine
GlcUA: glucuronic acid
CHSY-1: chondroitin sulfate synthase 1
CHSY-2: chondroitin sulfate synthase 2
CHSY-3: chondroitin sulfate synthase 3
CSGALNACT-1: chondroitin sulfates N-acetylgalacto saminyltransferase 1
CSGALNACT-2: chondroitin sulfate N-acetylgalactosaminyltransferase 2
C4ST-1: chondroitin-4-O-sulfotransferase 1
C6ST-1: chondroitin-6-O-sulfotransferase
ACAN: aggrecan
B4GALT-7: xylosylprotein beta 1,4-galactosyltransferase, polypeptide-7
XYLT-1: xylosyltransferase-1
HYAL-1: hyaluronidase-1
IL-1β: Interleukin-1β
TNF-α: tumor necrosis factor-α
TGF-β: transforming growth factor-β
DMMB: 1,9-Dimethylmethylene blue
SD: standard deviation
GAPDH: glyceraldehyde-3-phosphate dehydrogenase
