# Supplementary material for: Inflammatory microRNA-194 and -515 attenuate the biosynthesis of chondroitin sulfate during human intervertebral disc degeneration
Source: Oncotarget. 2017 May 2;8(30):49303–17. doi: 10.18632/oncotarget.17571 (PMC5564769; doi:10.18632/oncotarget.17571)
Supplement: Supplementary file 1 [file oncotarget-08-49303-s001.pdf]

Inflammatory microRNA-194 and -515 attenuate the biosynthesis of chondroitin sulfate during human intervertebral disc degeneration

Supplementary Materials

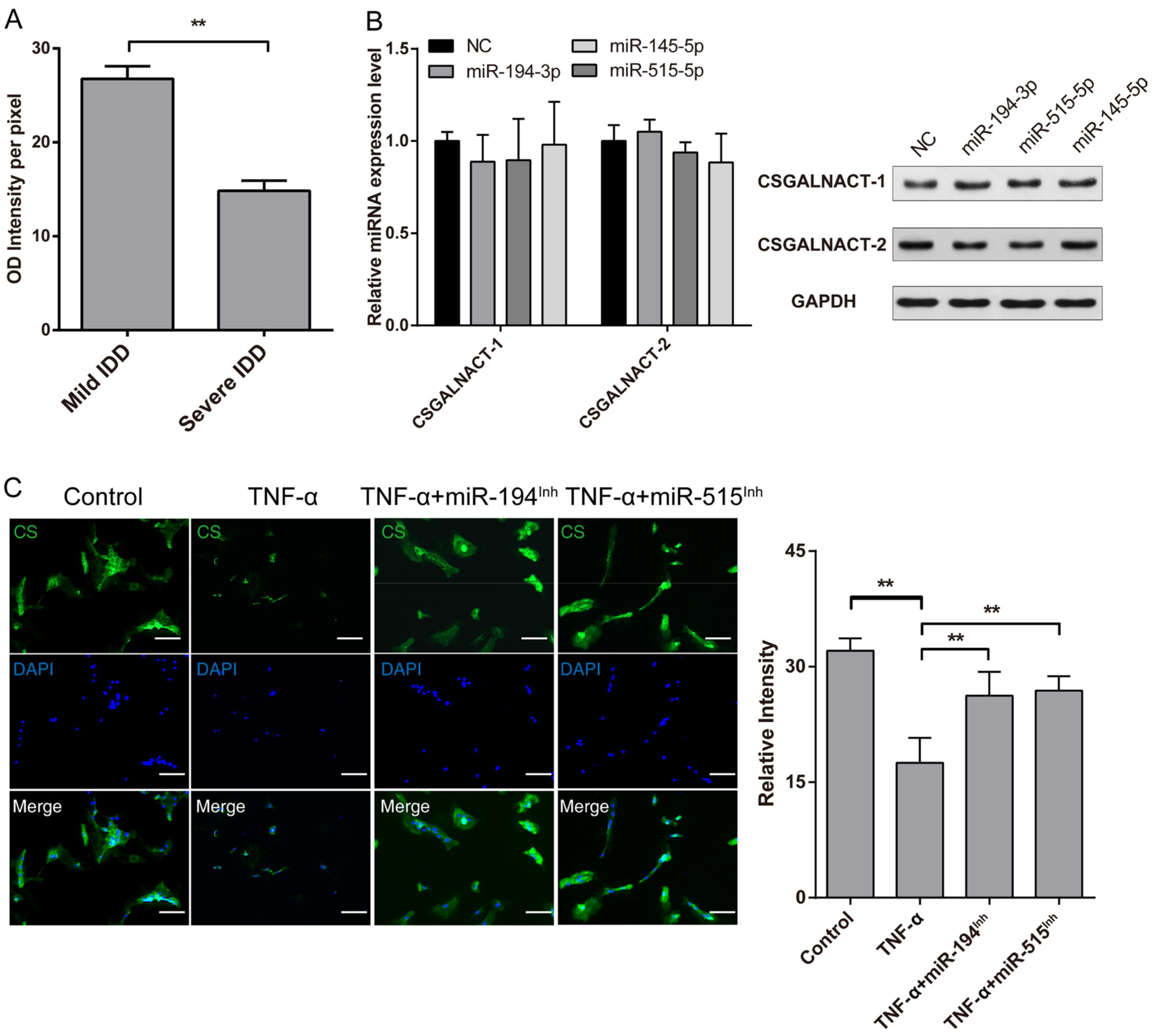

Supplementary Figure 1: (**A**) The quantification of relative intensity of CS Immunofluorescence microscopy between mild IDD NP and severe IDD NP samples. Data are shown as mean ± SD. $^{**}p < 0.01$. (**B**) Real-time PCR and Western blot analysis showing the expression level of CSGALNACT-1 and CSGALNACT-2 under miR-194, -515 or control miRNA mimic overexpression. Data are shown as mean ± SD. $^{**}p < 0.01$, $n = 3$, data were normalized to GAPDH. The right panel shows the relative protein level of CSGALNACT-1 and CSGALNACT-2. (**C**) Immunofluorescence images of CS in NP cells treated with 100 ng/ml TNF-α and miR-194 or miR-515 inhibitors or scramble control inhibitor for 72 h. The quantifications of relative OD intensity were shown in the right panel. Data are shown as mean ± SD. $^{**}p < 0.01$, $n = 5$, scale bars represents 50 μm.

Supplementary Table 1: Patients' information of the clinical samples

Patient number	age	Sex	Disc level	Duration of Symptoms (mo)	pfirrmann grade
1	25	F	L5/S1	23	2
2	30	M	L5/S1	13	2
3	35	F	L4/5	4	2
4	18	M	L4/5	6	2
5	27	M	L5/S1	17	2
6	25	F	L3/4	12	2
7	46	F	L5/S1	26	3
8	33	F	L3/4	22	3
9	60	F	L4/5	6	3
10	57	F	L4/5	24	3
11	58	M	L5/S1	12	3
12	48	F	L4/5	16	3
13	37	F	L5/S1	9	3
14	52	M	L4/5	17	3
15	53	M	L5/S1	18	3
16	37	M	L4/5	6	3
17	53	F	L5/S1	14	3
18	41	F	L4/5	24	3
19	78	F	L5/S1	33	4
20	53	F	L5/S1	12	4
21	41	M	L5/S1	29	4
22	42	F	L5/S1	16	4
23	66	F	L4/5	11	4
24	46	M	L5/S1	23	4
25	38	M	L5/S1	15	4
26	69	F	L4/5	26	4
27	40	F	L4/5	12	4
28	44	F	L5/S1	24	4
29	50	M	L4/5	6	4
30	42	M	L4/5	16	4
31	57	F	L5/S1	19	4
32	42	M	L4/5	7	5
33	56	M	L4/5	21	5
34	53	M	L3/4	11	5
35	61	F	L4/5	19	5
36	67	F	L5/S1	28	5
37	35	M	L2/3	-	1
38	58	F	L1/2	-	1
39	45	F	T12/L1	-	1
40	41	M	L1/2	-	1
41	36	M	L2/3	-	1

Supplementary Table 2: Primer sequences

Gene	Forward (5′→3′)	Reverse (5′→3′)
CHSY1	AAGTTCCCAGTGACAACCA	CACCCACTCGGACAAGAT
CHSY2	ACCTGGACAAGTATGAGTG	TACTGCTGTTTAGCGATC
CHSY3	ATCGCTAAACAGCAGTAAG	CTCTAAGGCATTCACCAA
CSGALNACT1	CTTTGGATTTGGGATGAC	TGTGGAGATACTTGCGATA
CSGALNACT2	GTCTCACTCGCCATCCTG	ACCCTCCTCATCTTCTTGTT
XYLT1	GAGGCTATTCCGCAACTT	GACGGTCACGGTCACATT
HYAL1	ACAGACTGGAATAGTGGCATAA	GGATTGGCAAAGAGTAGGC
B4GALT7	CGGCTGTGCAATGGGATG	GCAGGTGGCGAAATGTCTT
C4ST1	TGGATGAGGACCACGAGC	CTTCAGGTTGGCGGAGAC
C6ST1	CAGGTGCGGTGGCTCATA	AGGCTGGTCTCCAACTCTT
ACAN	ACTCTGGGTTTTCGTGACTCT	ACACTCAGCGAGTTGTCATGG
IL-1β	CGGCCACATTTGGTTCTAAGA	AGGGAAGCGGTTGCTCATC
TNF-α	GCCGAGTCTGGGCAGGTCTA	GGAGGCGTTTGGGAAGGT
TGF-β	CCCTGGACACCAACTATTGC	TGCGGAAGTCAATGTACAGC
GAPDH	CCATGTTCGTCATGGGTGTGAACCA	GCCAGTAGAGGCAGGGATGATGTTC
